# Defining ovarian reserve to better understand ovarian aging

**DOI:** 10.1186/1477-7827-9-23

**Published:** 2011-02-07

**Authors:** Norbert Gleicher, Andrea Weghofer, David H Barad

**Affiliations:** 1 Center for Human Reproduction - New York, New York, NY, USA; 2 Foundation for Reproductive Medicine, New York, NY, USA; 3 Department of Obstetrics, Gynecology and Reproductive Sciences, Yale University School of Medicine, New Haven, CT, USA; 4 Department of Obstetrics and Gynecology, University of Vienna School of Medicine, Vienna, Austria; 5 Department of Epidemiology and Social Medicine, Albert Einstein College of Medicine, Bronx, NY, USA; 6 Department of Obstetrics Gynecology and Women's Health, Albert Einstein College of Medicine, Bronx, NY, USA

## Abstract

Though a widely utilized term and clinical concept, ovarian reserve (OR) has been only inadequately defined. Based on Medline and PubMed searches we here define OR in its various components, review genetic control of OR, with special emphasis on the *FMR1 *gene, and discuss whether diminished OR (DOR) is treatable. What is generally referred to as OR reflects only a small portion of total OR (TOR), a pool of growing (recruited) follicles (GFs) at different stages of maturation. Functional OR (FOR) depends on size of the follicle pool at menarche and the follicle recruitment rate. Both vary between individuals and, at least partially, are under genetic control. The *FMR1 *gene plays a role in defining FOR at all ages. Infertility treatments have in the past almost exclusively only centered on the last two weeks of folliculogenesis, the gonadotropin-sensitive phase. Expansions of treatments into earlier stages of maturation will offer opportunity to significantly improve ovarian stimulation protocols, especially in women with DOR. Dehydroepiandrosterone (DHEA) may represent a first such intervention. Data generated in DHEA-supplemented women, indeed, suggest a new ovarian aging concept, based on aging of ovarian environments and not, as currently is believed, aging oocytes.

## Background

Though by some suggested to be renewable [1], current dogma still holds that women are born with their complete oocyte pool for life [2]. It is mostly made up of primordial follicles, containing oocytes arrested in meiotic prophase I, and remaining quiescent until recruited into follicle maturation. How primordial follicles are activated to enter maturation is not well understood yet but reflects complex processes of bi-directional signaling between oocytes and surrounding somatic cells [3]. In the mouse an important recent paper by Reddy et al suggested that oocyte-specific deletion of Pten (phosphatase and tensin homologe deleted on chromosome 10) results in premature activation of the primordial follicle pool, leading to premature ovarian failure (POF)/primary ovarian insufficiency (POI) [4]. Whether human activation may follow a similar pathway remains to be seen

Recruitment is a steady process. Cohorts of resting primordial, also called non-growing follicles (NGFs), are consistently recruited though, ultimately, only one single oocyte usually reaches ovulation [5]. Over more than four months of follicle maturation randomly recruited follicles are progressively aligned into generational cohorts of maturing follicles. At random recruitment is, thus, converted into episodic maturation, ultimately leading into regular menstrual cycle patterns. Even when ovulation fails, as often the case in polycystic ovaries, by time of maturation arrest, follicle cohorts appear already mostly aligned in size and maturation stages.

Normal young ovaries usually manage conversion from anarchical recruitment to episodic cyclic maturation well. Older and more dysfunctional ovaries, however, no longer do. Increasing dysfunction in alignment will, therefore, cause increasingly inhomogeneous follicle cohorts, entering the gonadotropin-sensitive stage of folliculogenesis. Older women, therefore, demonstrate wider oocyte maturation ranges than normal functioning, younger ovaries [6].

The importance of this follicle alignment process has not been well appreciated. Historically, fertility treatments, almost exclusively, depended on the gonadotropin-sensitive last two weeks of follicle maturation before ovulation. Practically all clinical and pharmacologic research has been directed at these two weeks, when alignment is already completed.

This manuscript proposes that improvements in current ovarian stimulation protocols, and fertility treatments in general, require a redefinition of what constitutes a complete "treatment cycle." Such reconsideration begins with the acknowledgment that follicle cohorts, entering their gonadotropin-sensitive phase, are products of already months-long maturation, taking place within distinct, age-specific ovarian environments, which are subject to dramatic changes as women age. Only treatments directed at these earlier stages of folliculogenesis will further improve ovarian stimulation.

### What is ovarian reserve (OR)?

Ovarian reserve (OR) is a widely used term that has largely remained undefined, and, to some degree, even misused. What is generally referred to as OR, really represents only small components of total ovarian reserve (TOR). A woman's cumulative hypothetical pregnancy chance is mathematically reflected in her complete follicle pool, her TOR. Since TOR declines with age [2], "ovarian age" is another frequently heard term to describe a woman's remaining reproductive capacity.

TOR mostly consists of NGFs (largely primordial follicles) and to a lesser degree of maturing growing follicles (GFs) after recruitment. But only the latter reflect the so-called functional OR (FOR), referred to in the literature, when the acronym OR is used. Concomitantly, when the acronym DOR is used, the meaning is to refer to diminished FOR.

Over time follicle recruitment diminishes TOR. In aging women, FOR, in parallel, declines reasonably predictably, and in age-specific boundaries [7,8]. Normal physiologic ovarian aging (NOA) is, thus, defined by age-specific declines of FOR within expected ranges.

Approximately 10% of women deviate from age-specific standards [9] and, before reaching menopause, are assumed to suffer from premature ovarian aging (POA) [8], also called occult primary ovarian insufficiency (OPOI) [10]. NOA and POA/OPOI share many characteristics but differ in others (Table 1): The size of a woman's initial follicle pool between birth and menarche is of great importance because it reflects the symbolic starting point of follicle depletion (though considerable depletion, of course, occurs already *in-utero*.) Published OR models demonstrate that, due to genetic preprogramming, pools vary greatly in size [11,12]. Wallace and Kelsey, suggest between 35,000 and 2.5 million follicles (average 295,000) per ovary at birth, and significantly smaller numbers by time of menarche [12].

**Table 1:** Characteristics of ovarian aging

Characteristics of ovarian aging	References
Varying initial oocyte numbers between individuals at birth/menarche	[11,12]
Varying pace of follicular recruitment between individuals	[12]
Decreasing pace of follicular recruitment with advancing age	[12]
Decreasing numbers of follicles in folliculogenesis with advancing age	
Increasingly poor egg quality with advancing age	[13-15]
*Due to decreasing follicles in folliculogenesis and poorer egg quality:*	
Decreasing embryo quality with advancing age	
Decreasing spontaneous fecundity with advancing age	
Decreasing oocyte numbers in IVF with advancing age	
Decreasing embryo numbers in IVF with advancing age	
Decreasing pregnancy rates with IVF	[7,8,15-18]
Decreasing pregnancy rates with infertility treatments in general	[19]
Increasing aneuploidy with advancing age*	[20] - [22]

*Women with POA/OCPOI, though demonstrating all other characteristics of NOA, at prematurely young ages still demonstrate normal, age-appropriate aneuploidy rates [26]. While clinically in all other characteristics exhibiting characteristics of "older" ovaries, embryo quality will still be relatively good, allowing for excellent pregnancy chances with IVF

Recruitment rates also appear to vary: The same model suggests a wide range of 100 to 7,500 follicles per month entering maturation and growth, with peak numbers reached at approximately age of 14. Thereafter, recruited follicle numbers persistently decrease, irrespective of original follicle numbers [12]. Combined, starting follicle numbers at menarche and follicle recruitment rates thereafter, therefore, determine remaining TOR and number of recruited follicles at all ages. Once recruited into maturation, follicles become GFs.

### Why all of this matters

Recruitment after menarche inversely correlates with remaining TOR [3]. Like starting follicle numbers, recruitment rates are also genetically preprogrammed. Genetics at all ages, thus, play a dominant role in determining TOR (for further detail, see *FMR1 *discussion below).

Fecundity (spontaneous conception chance) and fertility treatment success depend on TOR, and especially FOR: The lower FOR, the poorer are overall chances of conception [2,5]. As TOR and FOR decline with advancing age, pregnancy chances, therefore, decline in parallel.

Table 1 summarizes what defines normal ovarian aging (NOA): As recruited follicle cohort sizes decline, fewer follicles enter maturation, producing fewer preovulatory oocytes [12]. In parallel, oocyte quality declines [13-15], leading to smaller oocyte yields and poorer oocyte quality with in vitro fertilization (IVF) [7,8,15-18], poorer IVF pregnancy rates [16,18] and lower pregnancy rates after infertility treatments, in general [19]. In addition, embryo aneuploidy [20-22] and miscarriage rates [23,24] increase, ultimately resulting in poorer delivery rates after spontaneous pregnancies, and pregnancies following treatment [25].

Except for age-specific aneuploidy rates [26], all of these NOA characteristics are also shared by POA/OPOI. This one difference represents, however, a principal reason why pregnancy rates in POA/OPOI patients are usually higher, even if objectively measured FORs are similar [27].

### The clinical relevance of defining OR correctly

Since most GFs are on the way towards degeneration and apoptosis, only still unrecruited primordial (NGFs) really represent the true remaining TOR [11,12]. A clinical tool to assess NGFs does not exist. GFs, which are routinely assessed in clinical practice, and often erroneously referred to as OR, really reflect only a relatively tiny fraction of all follicles. It is only that small fraction of follicles that is clinically assessed by follicle stimulating hormone (FSH), anti-Müllerian hormone (AMH) and antral follicle counts (AFCs). We, therefore, in clinical practice only assess FOR, and a very short time sequence in a woman's follicle maturation.

Currently available ovarian assessment tools, therefore, only assess GFs or FOR. Likely still present unrecruited primordial follicles, if recruited, could offer significant additional pregnancy chance. They, however, remain unmeasured. Potential therapeutic opportunities for women with diminished FOR, therefore, appear obvious!

### Clinical utility of OR assessments

FSH, AMH and AFCs offer information on somewhat varying follicle populations within GFs. For example, post-primordial pre-antral, small follicles are, likely, best reflected in AFCs and by AMH, while larger gonadotropin-sensitive follicles are best represented by FSH [28-31]. Grynberg et al, however, recently reported that, in combination, large follicles show much poorer correlation to FSH and inhibin B than smaller antral follicles <7 mm size [32].

Potential differences in specificity are clinically important: For example, AMH appears more specific than FSH in predicting oocyte yields [33-35] and pregnancy chances [33-37]. This should not surprise since smaller pre-antral and antral follicles, which strongly associate with serum AMH concentrations, represent a majority of GFs. Antral follicles to a degree, however, also affect FSH [32]. Large differences in specificity between these two OR assays can, therefore, not be expected, reflected in relatively good overall clinical correlations between FSH and AMH assessments [38].

Which assays are utilized assumes more importance at their limits of sensitivity. For example, the AMH ELISA, in service at our center, defines undectable as <0.1 ng/mL [39]. Like abnormally high FSH (>15.0-20.0 mIU/mL), most fertility centers currently consider very low AMH (<0.8 ng/mL) diagnostic of severe DOR and, therefore, a contraindication to infertility treatments with use of autologous oocytes [40]. In such situations patients are, thus, denied treatment based on only GF assessments, and irrespective of TOR.

**Figure 1:**
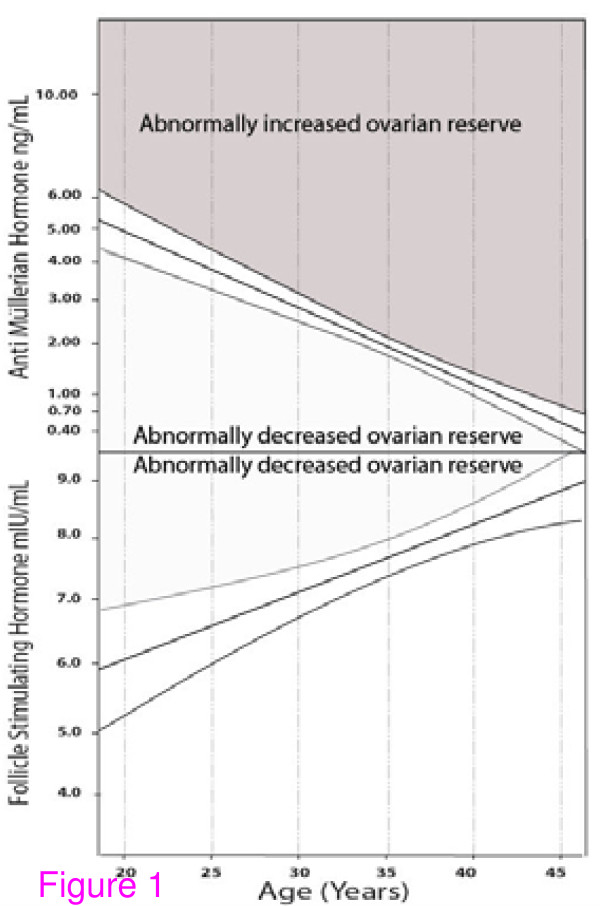
**Age-specific FSH and AMH levels: (Modified from Barad *et al*., 2010, with permission)**. The Figure presents on exponential scale age-specific AMH (ng/mL) and FSH (mIU/ML) in a center-specific patient population of oocytes donors and infertility patients. Modified with permission [7].

Assessments are currently at their best in young to middle aged women with normal age-specific OR, where, clinically, they are needed the least. Figure 1 demonstrates the 95% confidence intervals (CIs) of FSH and AMH at different ages. Both demonstrate narrowest ranges at approximately 32 to 33 years and the widest CIs at youngest and oldest ages [7]. Similar observations of decreasing specificity for FSH and AMH at extreme ages were also made when discrepancies between FSH and AMH were investigated in age-specific fashion [41]. While FSH and AMH, thus, in principle correlate well [38], differences between these two OR assays can be observed in individual patients, which then do have clinical significance [41].

We previously noted that recruitment rates inversely relate to TOR [3]. Elevated FSH and abnormally low AMH, therefore, do not preclude continuous potential availability of even substantial unrecruited NGFs. Poor GF numbers, reflecting poor FOR, therefore, do not suggest absence of all follicles.

Even POF/POI, and in physiologic menopause ovaries still contain substantial numbers of NGFs. A form of POF/POI, characterized by steroidogenic cell autoimmunity, demonstrates almost uniformly preserved follicle pools on ultrasound [28]. Indeed, in the past considered a rare finding, follicles can be seen on ultrasound in over a third of POF/POI cases [42]. Women with physiologic, age-appropriate menopause also almost uniformly still demonstrate follicles in their ovaries [11,12,43]

To contribute functionally, NGFs, however, also must be recruitable. Medications with ability to regulate follicular recruitment, therefore, have the potential of revolutionizing fertility treatments. Here is one, potentially already available, small example: If FSH, indeed, as reported recently, is also able to affect recruitment of primordial follicles [44,45], long-term, uninterrupted FSH exposure may, cumulatively, result in superior ovarian stimulation results to intermittent one-cycle stimulations, which have been clinical "dogma" for decades. In women with severely diminished ovarian reserve we, indeed, have preliminary evidence that this may be the case (Gleicher N and Barad DH, unpublished data). Specific medications with abilities to either down-regulate recruitment (for example with polycystic ovaries) or up-regulate recruitment (with low FOR) could then be the next development stage in fertility medications.

### Controlling OR

Wallace and Kelsey recently reaffirmed that ovarian aging varies between individuals [12]. Using age at menopause as end points, they determined that speed of follicle recruitment and follicle numbers vary significantly at different stages of life. We have come to similar conclusions, recently describing effects of the *FMR1 *gene on the ovary [46].

Evaluations of the *FMR1 *gene have become increasingly popular because of the gene's neuro/psychiatric effects [47]. It, however, also, independently, demonstrates specific ovarian effects [46]. Increased risk for POF/POI in women with premutation-range (ca. 55-200) CGG triple nucleotide repeats has been known for decades [47] but that even lower CGG repeat numbers may also denote risk towards premature ovarian senescence, at times representing milder forms of so-called POA/OPOI, is a more recent discovery [48-51].

The genotypes relating to ovarian function are distinctly different from the genotypes historically reported to define the gene's neuro/psychiatric risks. In regard to ovarian function, 26 to 34 CGG nucleotide repeats represent normal (median 30), independent of ethnicity/race [49]. Using this range to define genotypes, women can be designated as *normal (norm)*, when both alleles are in normal range, *heterozygous (het) *if one is normal and the other abnormal and *homozygous (hom) *if both alleles are outside normal range.

**Figure 2:**
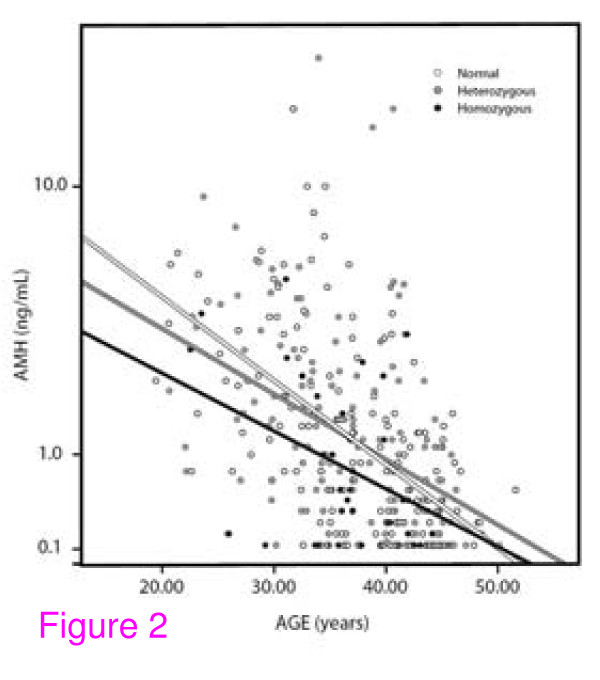
**Linear regressions of OR with advancing age (reflected by AMH) based on *FMR1 *genotype**. The figure depicts on logarithmic scale linear regressions of AMH (ng/mL) with advancing age in different *FMR1 *genotypes. Modified with permission from [46], where statistical differences between the three genotypes are presented in detail and where in age-binned analysis it is demonstrated that women with *norm *genotype decline precipitously in AMH around age 32, while *het *and *hom *genotypes demonstrate a slow, gradual decline).

Figure 2 demonstrates linear regressions of AMH over age, depending on whether women are *norm*, *het- *abnormal or *hom- *abnormal: Depending on *FMR1 *genotype, ovarian aging patterns differ. Before physiologic ovarian aging significantly contributes to OR at young ages, differences are most obvious. As expected, *norm *women demonstrate better OR than *het *females, with *hom *women demonstrating the lowest OR.

Differences between these three *FMR1 *genotypes persist with advancing female age, and take interesting, and somewhat surprising, turns: As Figure 2 demonstrates, the three genotypes do *not *age in parallel, as suggested by *FMR1*-independent models of Wallace and Kelsey [12] and Faddy and co-workers [43]. While *norm *women start with highest FOR, they quickly deteriorate and, by approximately age 35, cross the FOR regression line of *het *females, who initially had started out with lower FOR. In the late 40s the FORs of *norm *women then also fall below those of *hom *females.

These distinct "ovarian aging" patterns strongly imply that *FMR1 *genotypes define speed of follicular recruitment and, inversely, rates of decline in OR. If, as previously discussed, recruitment rates, indeed, reflect TOR, this observation also reflects that *FMR1 *genotypes, likely, reflect TOR. *FMR1 *can, therefore, be viewed as an "ovarian aging gene."

*Norm *women at younger ages appear to recruit actively and, therefore, likely deplete TOR quicker than *het *and *hom *females, who from young age on recruit at much slower pace. The latter two genotypes, therefore, demonstrate much slower and steadier declines in AMH levels. Still preliminary data suggest that *het *patients can be further subdivided into *het-norm/high *and *het-norm/low *sub-genotypes (depending on whether the abnormal allele is abnormally high or low), which may further differ in respective aging curves [52,53].

These observations confirm statistical associations between *FMR1 *genotypes (based on normal CGG range of 26 to 34) and FOR, based on FOR assessment with AMH. They, thus, suggest significant relevance of triple CGG repeat counts to clinical practice [49,50], and that *FMR1 *genotypes, within reasons, already at young ages allow predictions about "ovarian aging" patterns. Paradoxically, due to rapid recruitment at young ages, the *norm *population appears at greatest risk for early follicle depletion and, possibly, early menopause. Whether early depletion can be equated with early menopause has, however, so far not been established.

*FMR1*, thus, is involved in regulating FOR but may also affect TOR by regulating follicular recruitment. How *FMR1 *actually regulates physiologic functions has remained controversial: In association with neuro/psychiatric consequences, the number of CGG triple repeats is a determining factor. Risks increase with increasing expansion sizes up to approximately the lower half of the so-called premutation range (ca. 55-200 repeats), though not necessarily in linear fashion [47,54]. The gene codes for an RNA-binding protein, the so-called fragile X mental retardation protein (FMRP) [55], important in synaptic physiology and apparently demonstrating RNA toxicity [56].

Chen and associates reported that maximal translation of the gene product and that the switching point between positive and negative effects of the gene occurs at 30 CGG repeats [57]. This exactly reflects the median of the normal range, reported for the gene's ovarian function [46,58].

This normal range of 26 to 34 repeats also contains at midpoint the tall distribution peak of CGG repeats in the general population, reported by Fu and associates at 29 to 30 repeats [59]. Combined, these observations suggest that FMRP, the gene product of *FMR1*, and/or its translation, may play a role in follicular recruitment and "ovarian aging."

Mouse models have allowed progress in understanding follicular recruitment and, by extension, OR regulation. We noted earlier the important recent paper by Reddy et al [4]. In humans, this area, however, still largely represents a black box [3], resulting in many models of "ovarian aging" [11,12,43] but little factual data. Described *FMR1 *genotypes raise additional questions about these proposed human models of ovarian aging normal ovarian aging, quite obviously, has to be defined separately for individual *FMR1 *genotypes.

In some aspects, Wallace and Kelsey's model [12] appears superior to others since it detected rapid declines in OR at younger ages than Faddy and co-workers, who reported an accelerated declines only at age 37 to 38 years [43]. Considering varying "ovarian aging" patterns of different *FMR1 *genotypes [46], Faddy and Gosden's later timing, however becomes understandable as *norm *women demonstrate accelerated declines in FOR at approximately age 35 years (Figure 2), while *het *and *hom *females demonstrate a more gradual decrease. Mathematically combined, all three genotypes, indeed, resemble Faddy and Gosden's model.

Ultimate purposes of *FMR1 *genotypes remain to be determined. Since *norm *women, in contrast to *het *and *hom *counterparts, relatively quickly deplete FOR, the latter two genotypes preserve better FOR into advanced age. One can speculate that, due to better FOR at older ages, especially *het*, but also *hom *women, may end up with higher spontaneous and treatment-induced pregnancy chances. Indeed, *norm *women may be the ones with earliest menopause, suggesting a need to reanalyze menopause data based on *FMR1 *genotypes.

*FMR1 *genotypes may, finally, also explain why a gene with such severe neuro/psychiatric and reproductive consequences is, nevertheless, so highly preserved. The answer may lie in survival of the species: By expanding a relatively narrow fertile window at young ages in *norm *women to more advanced ages in *het *and *hom *women, a longer window for reproductive success is opened and, with it, higher likelihood for preservation of the species.

### Diagnosis and treatment

Since it is generally believed that FOR declines with age, it is somewhat peculiar that evaluations are still mostly based on age-independent FSH, AMH and AFCs. As FOR declines, these three OR parameters, of course, change in parallel [7,8]. To define DOR, independent of age, is, therefore, limiting.

We established age-specific cut off values for our patient population, based on 95% CIs for FSH [8] and AMH [7] (Figure 1), and demonstrated their superiority in predicting DOR [7,8]. Universally applicability age-specific values appear overdue.

Practically all women develop diminished FOR above age 40, as their ovaries age [8]. Age-specific testing is, therefore, primarily useful in younger women, where diminished FOR is frequently overlooked and, often, mistaken for so-called unexplained infertility [60]. Timely FOR evaluations are especially importance in young women at risk for POA/OPOI. Risk factors include *FMR1 *genotypes [46], history or family history of autoimmunity [61-63], history of ovarian surgery [64], chemo/radiation therapy [65] and maternal history of early menopause [66].

With accurate diminished FOR diagnosis still a rather imprecise science, treatments are limited and, often, controversial. Indeed, controversies abound: For example, do increasing gonadotropin dosages improve oocyte yields? Many authorities believe that dosages beyond 225-300 IU are useless [67,68]; others disagree. We reported that increasing stimulation benefits women with POA/OPOI, but to lesser extent women with NOA [69]. Both, of course, represent distinctively different pathophysiologies [70].

Later stage GFs from large pre-antral to pre-ovulatory stages are gonadotropin (FSH) - sensitive [71]. Contradicting older reports that earlier stage follicles are unaffected by FSH [72], more recent data demonstrate that FSH also significantly affects primordial and early antral follicles [44,45]. We, therefore, previously pointed out that continuous FSH exposure may beneficially affect follicle recruitment. The same, of course, may also apply to higher dosages. There is, however, one difference: Since recruitment of currently gonadotropins-sensitive cohorts occurred months earlier, effects of currently applied higher gonadotropin dosages would, clinically, likely, be comperatively insignificant.

Any observed improvements in oocyte yields, therefore, have to be consequence of reduced follicle degeneration and apoptosis (i.e., follicle "rescue"). Based on Henderson and Edwards' "production line hypothesis," higher gonadotropins dosages then, indeed, should be more likely successful in younger women's ovaries [73].

Quality of ovarian stimulation has remained as controversial as quantity: Agonist or antagonist [74,75], pure FSH or human menopausal (hMG) stimulation [76,77]. Which amongst those offers better results in diminished FOR patients has so far remained unresolved.

Our center primarily utilizes a microdose agonist protocol, with FSH preponderance and hMG contribution [78]. In younger women an FSH protocol may also be effective [79]. A microdose agonist protocol was also proposed by Schoolcraft and associates [80], with both protocols attempting to avoid suppressive effects on ovaries. Others see no difference between microdose agonist and antagonist protocols [81,82]. Appropriately controlled studies are lacking.

### Dehydroepiandrosterone and a new concept for ovarian aging

One of the most controversial issues in reproductive medicine is, however, undoubtedly, the questions whether diminished FOR can be pharmacologically improved. This may be a principle reason why dehydroepiandrosterone (DHEA) supplementation [83,84], though now utilized by approximately one third of IVF centers world-wide (http://www.ivf-worldwide.com), is not used even more widely.

DHEA supplementation improves egg/embryo quantity/quality and pregnancy chances [85]. Remarkably, spontaneous miscarriage rates are also decreased to levels in normal, fertile populations [86]. Since diminished FOR patients, likely, demonstrate the highest spontaneous miscarriage rates amongst infertility patients [23,24,87] such profound declines can mathematically not be achieved without decreasing aneuploidy rates. And DHEA supplementation has, indeed, been demonstrated to lower aneuploidy [88,89].

DHEA improves pregnancy outcomes even in women with most severe degrees of diminished FOR, who have no, or only minimal FOR left, with undetectable to extremely low AMH (<0.4 ng/mL) [90]. Except for our published case series [91], the literature contains only one published case of pregnancy with undetectable AMH [90]. Following DHEA supplementation, we recently reported over 30 pregnancies in women with either undetectable or extremely low AMH, reaffirming very low miscarriage rates even in most severe diminished FOR [91]. Since publication of this study the number of pregnancies in such patients has almost doubled.

While, even with DHEA supplementation, pregnancy and delivery chances in severe diminished FOR patients may be low, they are not as low as often suggested [91]. One, therefore, should be cautious about withholding care to patients because of allegedly too poor FOR [40].

It also appears time to abandon healthy skepticism about DHEA. Since prospectively randomized studies in women with severe diminished FOR are difficult to conduct (two such studies had to be abandoned because FOR patients objected to randomization) [85], skeptics have been unwilling to accept other study formats as best available evidence. Israeli investigators, however, recently published a first, small prospectively randomized DHEA study, confirming the hormone's efficacy with diminished FOR [92].

Minimal or even undetectable AMH, thus, does not preclude pregnancy with DHEA. This does not surprise because, as long as ovaries contain follicles, at least theoretically, pregnancies should be possible. Even menopausal ovaries still contain approximately 1,000 follicles in freshly menopausal women [11,12,28,42,43]. Assuming these follicles, and their oocytes, are functionally intact, recruitable and given opportunity to go through normal follicle maturation, why should they not be able to lead to pregnancy?

Then why don't they?

We noted at the beginning that current dogma still holds that women are born with their life-long follicle/oocyte pool. Dogma further holds that these oocytes "age" as women age, leading to progressively declining oocyte quality with advancing female age. Decreasing oocyte quality is irreversible and, in turn, results in increasing aneuploidy, lower implantation rates, declining pregnancy chances per embryo, and increasing spontaneous miscarriage rates. In short, aging oocytes represent the principal culprits in ovarian aging [5,93].

If correct, one expects aging oocytes to be progressively more damaged, very likely an irreversible process. Incidentally occurring pregnancies, at very advanced ages or with severe diminished FOR, can easily be attributed to genetic variations in women's fecundity [94] or mere chance. A concept of aging oocytes, however, does not explain why, after DHEA-supplementation, miscarriage rates in even the most severe diminished FOR cases remain very low, resulting in surprisingly high live birth rates [91].

In the presence of irreversibly deteriorating oocyte quality, such outcomes should be unachievable since already damaged oocytes only very unlikely can still be "rescued" or pharmacologically "rejuvenated." A direct effect of DHEA on oocytes, therefore, appears unlikely. DHEA, instead, probably has different targets, and effects DHEA may exert on oocyte quality have to precede significant oocyte damage. This means that these effects are, likely, indirect, and occur at relatively early stages of follicle maturation.

If DHEA does not directly target oocytes, yet oocyte quality still improves, only one possible explanation remains: The target of DHEA has to be the ovarian environment in which follicles mature. DHEA in some ways improves the ovarian environment, leading to better follicle maturation and, therefore, better oocyte quality as end product.

Under such a concept unrecruited oocytes in primordial follicles, however, can*not *age, as current dogma holds. As long as unrecruited, they have to maintain their original quality. Only once recruited and entering maturation do they risk "losing quality" if their maturation takes place within a poor quality ovarian environment. Since ovarian environments decline in quality as women age, aging women produce increasingly poor oocytes.

Building on such a concept of aging ovarian environments, above described DHEA effects on ploidy and miscarriage rates, suddenly, are not only plausible but outright logical. DHEA levels decline significantly with advancing age in even healthy women and men [95] and, often undiagnosed, with adrenal insufficiency [96].

Potential therapeutic effectiveness of DHEA (and other androgens) in DOR is also strongly supported by recent mouse studies, which demonstrate the essential importance of ovarian androgens to normal follicle development and female fertility [97].

Hodges and associates, already a number of years ago, suggested that ovarian environments may be subject to therapeutic interventions, which could reduce spindle defects during meiosis, leading to lower aneuploidy and miscarriage rates [21]. Female aging, of course, predisposes oocytes towards meiotic nondisjunction [98].

DHEA, therefore, may be a first restorative medication for aging ovarian environments, with others likely to follow. Other candidates have already been suggested: Based on loss of mitochondrial function with advancing age, Casper's laboratory in Toronto, Canada, proposed supplementation with the mitochondrial nutrient coenzyme Q10 (CoQ10). These authors demonstrated favorable effects on ovarian reserve in a mouse model [99]. A mitochondrial DHEA effect cannot be ruled out since androgens beneficially affect mitochondrial function [100]. Mitochondrial DNA content, in general, has been reported low in women with DOR [101]. Korean investigators reported that co-administration of leptin with gonadotropins regulates angiogenesis and improves ovarian response and oocyte quality in aged mice [102].

A new concept of aging ovarian environments, rather than aging oocytes, offers considerable new opportunities for treatment of diminished FOR, allowing for successful fertility therapy into older age and closer to menopause. For developed countries like the United States, where women above age 40 are the most rapidly growing age group having children [40], this could have considerable impact on public health.

Over the last 50 years, most research in the specialty has almost exclusively concentrated on the gonadotrpin-sensitive stages of folliculogenesis. Over the next decades the earlier phases of follicular maturation deserve closer attention.

## Abbreviations

AFC: Antral follicle count; AMH: Anti-Müllerian hormone; CIs: Confidence intervals; CoQ10: Coenzyme Q10; DHEA: Dehydroepiandrosterone; DNA: Deoxyribonucleic acid; DOR: Diminished ovarian reserve; FMRP: Fragile X mental retardation protein; FSH: Follicle stimulating hormone; FOR: Functional ovarian reserve; GFs: Growing follicles; *het: *heterozygous; hMG: Human menopausal gonadotropin; *hom: *homozygous; IVF: In vitro fertilization; NGFs: Non-growing follicles; NOA: Normal physiologic ovarian aging; *norm: *normal; OPOI: Occult primary ovarian insufficiency; OR: Ovarian reserve; PCO: Polycystic ovaries; POA: Premature ovarian aging; POF: Premature ovarian failure; POI: Primary ovarian insufficiency; TOR: Total ovarian reserve

## Competing interests

N.G and D.H.B. are listed as co-inventors on already awarded or still pending U.S. patent applications, which claim therapeutic benefits from DHEA supplementation in women with diminished ovarian reserve, and diagnostic benefits from determination of CGG nucleotide repeats on the *FMR1 *gene. All three authors received in the past research support, travel funds and speaker honoraria from various pharmaceutical companies, though none related in any way to in this manuscript covered topics.

## Authors' contributions

N.G. and D.H.B contributed equally to this manuscript by being involved in design of the project, data analysis and manuscript preparation. D.H.B contributed more to statistical data evaluations and N.G. to manuscript preparation. A.W. contributed to conceptual design. All authors approved the final manuscript. All authors read and approved the final manuscript.
